# 

**Published:** 1904-06-15

**Authors:** 


					﻿THE
DENTAL REGISTER
EDITED BY
NELVILLE S. HOFF, D. D. S.,
ANN ARBOR, MICH.
Vol. LV1II.	JUNE 15, 1904.	No. 6.
PRICE, ONE DOLLAR A "YEA RIH ADVANCE.
PUBLISHED MONTHLY BY
SAMUEL A, CROCKER & CO.,
THE OHIO DENTAL AND SURGICAL DEPOT.
15“ to 30 "W. ~ tl 1 St., Ciiiciiiiinti, O.
Entered at the Postoffice at Cincinnati, 0., as second-class mail matter.
				

